# Assessment of Automated Flow Cytometry Data Analysis Tools within Cell and Gene Therapy Manufacturing

**DOI:** 10.3390/ijms23063224

**Published:** 2022-03-17

**Authors:** Melissa Cheung, Jonathan J. Campbell, Robert J. Thomas, Julian Braybrook, Jon Petzing

**Affiliations:** 1 Centre for Biological Engineering, Loughborough University, Loughborough LE11 3TU, Leicestershire, UK; r.j.thomas@lboro.ac.uk (R.J.T.); j.petzing@lboro.ac.uk (J.P.); 2 National Measurement Laboratory, LGC, Queens Road, Teddington TW11 0LY, Middlesex, UK; jonathan.campbell@lgcgroup.com (J.J.C.); julian.braybrook@lgcgroup.com (J.B.)

**Keywords:** flow cytometry, automated data analysis tools, ATMP manufacturing, regulatory compliance

## Abstract

Flow cytometry is widely used within the manufacturing of cell and gene therapies to measure and characterise cells. Conventional manual data analysis relies heavily on operator judgement, presenting a major source of variation that can adversely impact the quality and predictive potential of therapies given to patients. Computational tools have the capacity to minimise operator variation and bias in flow cytometry data analysis; however, in many cases, confidence in these technologies has yet to be fully established mirrored by aspects of regulatory concern. Here, we employed synthetic flow cytometry datasets containing controlled population characteristics of separation, and normal/skew distributions to investigate the accuracy and reproducibility of six cell population identification tools, each of which implement different unsupervised clustering algorithms: Flock2, flowMeans, FlowSOM, PhenoGraph, SPADE3 and SWIFT (density-based, *k*-means, self-organising map, *k*-nearest neighbour, deterministic *k*-means, and model-based clustering, respectively). We found that outputs from software analysing the same reference synthetic dataset vary considerably and accuracy deteriorates as the cluster separation index falls below zero. Consequently, as clusters begin to merge, the flowMeans and Flock2 software platforms struggle to identify target clusters more than other platforms. Moreover, the presence of skewed cell populations resulted in poor performance from SWIFT, though FlowSOM, PhenoGraph and SPADE3 were relatively unaffected in comparison. These findings illustrate how novel flow cytometry synthetic datasets can be utilised to validate a range of automated cell identification methods, leading to enhanced confidence in the data quality of automated cell characterisations and enumerations.

## 1. Introduction

Flow cytometry is a single-cell analytical technique widely applied within manufacturing of advanced therapy medicinal products (ATMPs) and tissue engineered products to measure cell product characteristics, in accordance with Good Manufacturing Practice (GMP) and quality guidelines laid out by regulatory authorities such as the European Medicines Agency (EMA) and the US Food and Drug Administration (FDA) [1,2].

Typical ATMP drug product critical quality attributes (CQAs) evaluated by flow cytometry include identity, purity, potency, quantity, and viability [3]. These CQAs are usually measured from initial sample reception, and at each substantial manipulation step until final characterisation for product release.

Since flow cytometry plays a critical role in ATMP manufacture, the need for continual development of best practice, along with standardisation within the field is well recognised [4]. For instance, the British Pharmacopoeia recently prioritised the preparation of authoritative guidance on the application of flow cytometry for its cell and gene therapy stakeholder communities [5]. Such guidance documents cover the major sources of variation in flow cytometry, these being: starting materials and reagents, equipment, sample preparation, and data analysis. The lattermost factor, data analysis (manual gating), is a significant source of variation, and its removal from the analytical process has been shown to reduce inter-laboratory variation by as much as 5–20% [6]. Manual setting of gates is difficult to reproduce, subjective [7,8], and potentially biased in favour of a value that aids product release and a successful manufacturing run.

The data analysis aspect of flow cytometry has rapidly evolved in recent years with the development of a wide array of automated cell population identification software that implement unsupervised and supervised machine learning algorithms [9]. These include tools for dimensionality reduction (e.g., t-SNE and UMAP), clustering (e.g., FlowSOM, SPADE and SWIFT), scaffold maps, trajectory inference, and classification and regression (reviewed in [10]).

Previous work from benchmarking studies have suggested certain automated methods were able to reliably replicate manual gating. For example, the FlowCAP competitions saw several algorithms (ADICyt, SamSPECTRAL, and flowMeans) score highly on accuracy in cell identification challenges [11]. Similarly, a comparison of clustering methods focussed on high-dimensional data identified FlowSOM, X-shift, PhenoGraph amongst others as high performing [12]. However, datasets used for these critical assessments often come from a limited range of cell or disease models. Efforts from the recently launched National Institute of Standards and Technology (NIST) Flow Cytometry Standards Consortium to develop biological reference materials, reference data and reference methods are not yet applicable to address the sources of variability from automated data analysis software [13]. Although uptake of these advanced automated data analysis tools within ATMP manufacturing is largely unknown, a recent survey of the clinical community suggests that 20% of clinical laboratories sometimes or usually use them [9], and this number is expected to increase as the toolsets available mature.

Related machine learning-based computational technologies intended for patient diagnostic, treatment or preventative purposes are authorised by the FDA under Software as a Medical Device (SaMD) regulations [14], with a further proposed Artificial Intelligence and Machine Learning (AI/ML)-based SaMD regulatory framework [15]. To date, it appears that no such software focussed on analysis of flow cytometry data have been approved [16]. Relevant ISO/IEC standards for SaMDs include IEC 62304:2006, which defines the life cycle requirements for medical devices software to ensure safety and effectiveness, and ISO 14971:2019, which establishes the application of risk management to medical devices [17,18]. New guidance and efforts to standardise AI/ML in health care are beginning to emerge [19,20], but there is a lack of specific regulatory guidance on their use in the manufacturing of medicines.

The biomanufacturing community faces a challenge on how to compliantly adopt automated flow cytometry data analysis software in ATMP biomanufacturing process controls. While these automated analysis tools have the potential benefit to improve the quality of ATMPs and the capacity to minimise operator variation, in many cases, confidence in these nascent technologies has yet to be fully established. Specifically, a gap currently exists in the toolsets available for standardisation and testing of automated flow cytometry data analysis methods, potentially leaving manufacturers unable to demonstrate a method is fit for its intended purpose, and limiting the trust and transparency of these software tools among users and regulators.

**Figure 1:**
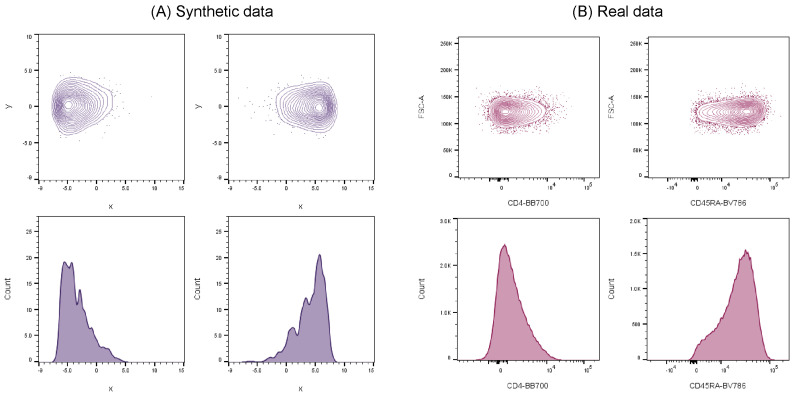
Inter-comparison between synthetic and real flow cytometry data showing clear similarities among cell distribution properties.

In order to address these issues, synthetic flow cytometry datasets have been previously developed to aid validation of automated cell population identification tools and were shown to successfully mimic real cell data [21]. These synthetic datasets demonstrated clear similarities in cell distribution characteristics when compared against real-world flow cytometry data (Figure 1), and therefore can be used as credible substitutes to represent actual experimental data.

Synthetic datasets are used in this research because: (1) they simplify the complexities of real-world data, enabling the separation of interacting factors that cloud the understanding and assessment of automated software, (2) they provide a ground truth that allows measurement accuracy to be explicitly determined (something that is difficult to achieve using real cell data), and (3) they overcome challenges in the acquisition of biological samples related to time, cost, scarcity of rare samples, and data privacy concerns.

Within the research reported here, the novel synthetic datasets containing controlled separation between clusters with normal or non-normal probability distributions are applied to a selection of flow cytometry computational tools that utilise different classes of clustering algorithms. We compare the performances of these different software using accuracy and repeatability evaluation metrics for showing trends in performance between software when analysing clusters with specific degrees of separation, and with skewed cell populations.

This research is intended to provide flow cytometry users in the biomanufacturing community with a better understanding of the characteristics, opportunities and limitations of automated data analysis software, ideally leading to enhanced confidence in the data quality of cell characterisations. In addition, it shows how a framework for benchmarking toolsets can be specifically designed for selection/validation of automated data analysis software.

## 2. Materials and Methods

### 2.1. Datasets

In order to perform a fair comparison between different automated data analysis software, synthetic flow cytometry reference datasets were designed and generated (as described in [21]). Out of the commonly recognised data characteristics or potential statistical attributes identified, we targeted the separation and the skew characteristics to control and modify in our datasets, because these properties had not been addressed in previous work and/or the designs had not been approached in a systematic manner. To retain the focus on these properties, non-target characteristics such as cluster sizes and the number of dimensions were kept constant, and the element of noise relating to real data was excluded.

#### 2.1.1. Separation Dataset

The purpose of these datasets was to evaluate software performance in identifying and partitioning cell populations as the clusters came close together.

Separation datasets were prepared using the R *clusterGeneration* package [22] with the following parameters:

- Number of clusters: 2 or 3,
- Cluster size: 1000 points per cluster,
- Separation index (SI) values: from −0.3 to +0.3, at 0.1 intervals, and
- Cluster covariance matrices: eigenvalues between 1 and 5.

This approach generated datasets with different degrees of separation between neighbouring clusters ranging from well separated to merged. Nine random normally distributed cluster replicates were generated at each SI value. Parameters were selected to give a range of variability in the diameter and shape of clusters similar to those seen in exemplar real flow cytometry data. Datasets were converted to FCS 3.1 format using the R package *flowCore* [23].

#### 2.1.2. Skew Dataset

The purpose of this skew dataset was to evaluate software performance in identifying and partitioning cell populations as the clusters displayed different levels of non-normal distributions.

Skew datasets were built in multiple stages. First, individual skew clusters were prepared with the function rmsn in the package *sn* [24], using the following parameters:

- Number of clusters: 1 (clusters later joined together),
- Cluster size: 1000 points per cluster,
- Mean vector: [0,0],
- Covariance matrix: values between 1 and 5, and
- α skew value: values between 2.5 and 10, at intervals of 2.5.

of which the α parameter regulated asymmetry. Likewise random cluster replicates were generated at each skew direction (left and right) along the *x*-axis.

During cluster generation, it was found that applying the skewing parameter (α) caused the diameter of the elliptical cluster to reduce along the *x*-axis. To compensate for this, clusters were elongated to obtain a pre-skew diameter using the R package *scales* [25]. The skewness of the clusters before and after rescaling were identical (measured using the R package *psych* [26]) determined by the asymmetry around the mean remaining unchanged.

Next, two clusters were joined together. A new level of complexity was introduced compared to normally distributed clusters because asymmetric clusters could be orientated in three ways: head to head, head to tail, and tail to tail (assuming the skew is introduced only along the *x*-axis). Clusters with the same α skew values were paired together (i.e., clusters with different skews were not combined).

Finally, one cluster was shifted further away from the other through vector operations in R. The distance between two clusters was measured with the *clusterGeneration* package [22]; datasets with a SI value between −0.25 and −0.15 were selected for further processing. Files were again converted to FCS 3.1 standard using *flowCore* [23].

This approach generated a library of two-cluster synthetic datasets in two dimensions with 1000 datapoints per cluster, with different levels of skew and skew–direction pairs.

### 2.2. Software Runs

The synthetic datasets were processed through six flow cytometry automated data analysis software (Table 1), each of which implement different unsupervised clustering algorithms: Flock2 [27] (via ImmPort Galaxy [28]), flowMeans [29], FlowSOM [30], PhenoGraph [31] (R implementation [32]), SPADE3 [33,34] and SWIFT [35,36].

It is recognised that many supervised techniques for automated cell population identification are available (e.g., FlowDensity [37]); however, these tools have not been included in this study because a significantly different approach in actual methodology of synthetic dataset design/application would be needed, mainly the need for extensive pre-training and training datasets containing meta-labelled data, and specific design of testing datasets.

### 2.3. Statistics and Performance Evaluation Metrics

Methods used for statistical analysis included the mean, sample standard deviation, and coefficient of variation (CV).

The software outputs were recorded, and the absolute difference between cell populations of cluster 1 to the reference value was calculated in percentage terms, as in Equation (1).

(1) $$\mathrm{Difference}\;\mathrm{to}\;\mathrm{reference}\;\%=\frac{\left|A-B\right|}{\mathrm{Total}\;\mathrm{events}}\times 100$$

where *A* is the reference cluster 1 count, and *B* is the software cluster 1 count.

## 3. Results

### 3.1. Output Number of Clusters

We assessed the performance of the six automated data analysis software, each of which implement different clustering algorithms to identify and quantify cells: Flock2, flowMeans, FlowSOM, PhenoGraph, SPADE3, and SWIFT (density-based, *k*-means, self-organising map, *k*-nearest neighbour, deterministic *k*-means, and model-based clustering, respectively).

We first investigated whether the software could partition the datasets to give the same number of clusters originally designed into them. We found that returning the desired number of clusters was straightforward for tools such as flowMeans, where the input number of clusters (*k*) directly determined the output. Obtaining the desired number of clusters from other software was more complex. In SWIFT, the input *k* served as an initial estimate which sometimes varied from the final output cluster number after subsequent cluster splitting and merging processing steps. In SPADE3, the default user settings automatically over-clustered the data into a minimum spanning tree with hundreds of nodes, with a subsequent ’semi-automated’ feature to suggest tree partitioning to the user. Here, the tree partitioning step was applied until the desired number of clusters were produced. PhenoGraph, and occasionally Flock2 and SWIFT, tended to over-cluster the data, so additional manual steps were performed to merge sub-clusters together.

In general, the manual workload increased in proportion to the number of clusters generated by a software above the desired amount, illustrating a paradox of increased human intervention in a supposedly automated process designed to reduce operator variation. We also found that flowMeans and FlowSOM did not permit outputs of two clusters, so processing of two-cluster datasets returned a minimum cluster number of three, thus again requiring a manual merging step.

Overall, strategies to obtain the desired output number of clusters varied significantly between different software, with some requiring repeated tuning of input parameters and/or post-clustering manual interpretation steps, suggesting a high level of operator training required, as opposed to casual use.

### 3.2. Clustering Characteristics

**Figure 2:**
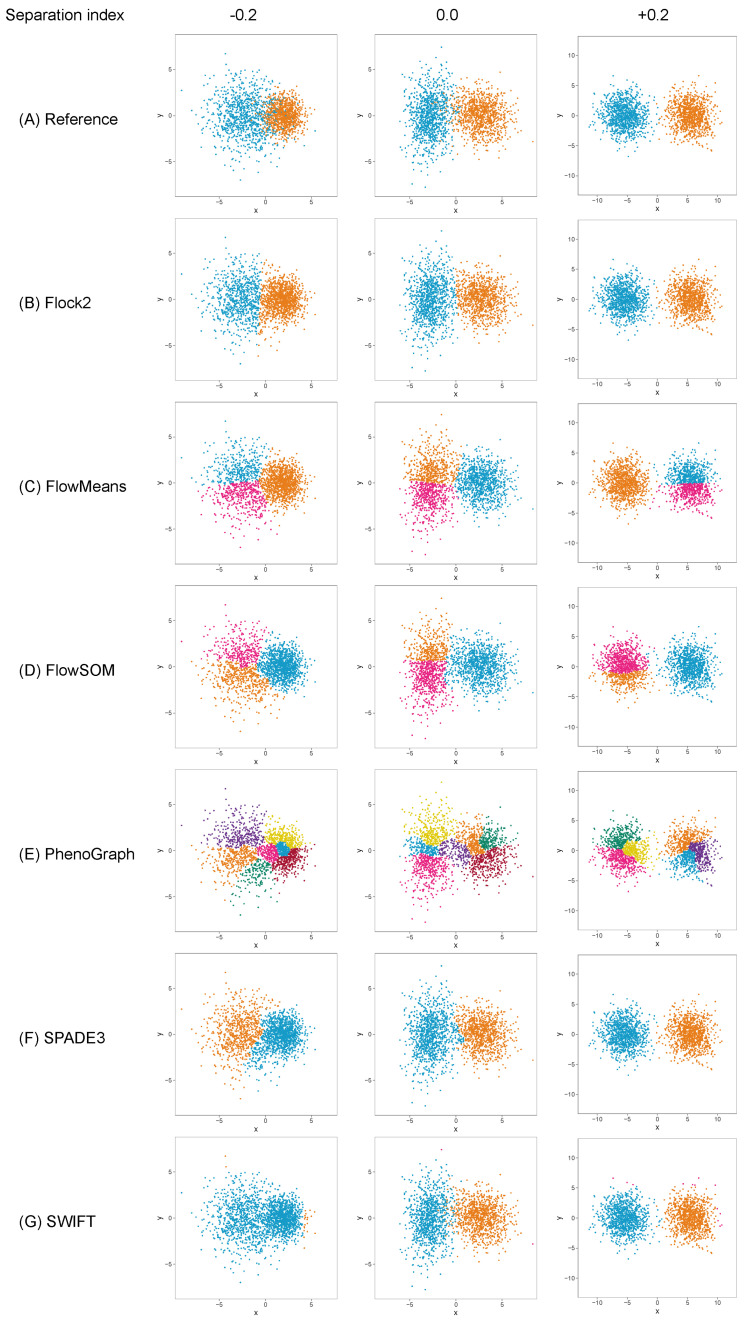
Clustering examplesfrom different software on a two-cluster synthetic flow cytometry dataset with different degrees of separation between clusters.

**Figure 3:**
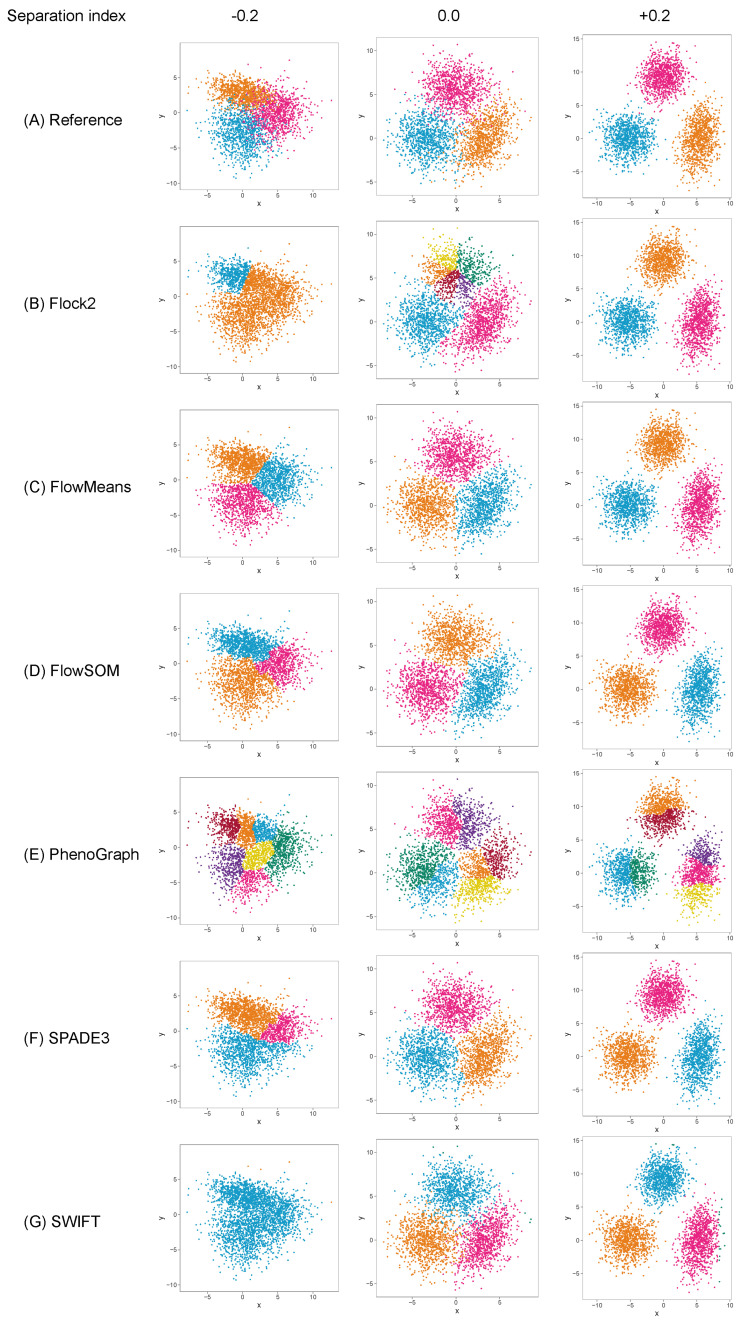
Clustering examplesfrom different software on a three-cluster synthetic flow cytometry dataset with different degrees of separation between clusters.

The different software tools tested here all utilised different clustering algorithms, and certain data partitioning characteristics became particularly noticeable with overlapping clusters as the data became unstructured. Reference cluster designs are depicted in Figure 2A and Figure 3A, along with the raw software clustering outputs, before manual intervention was performed to merge sub-clusters together from, e.g., Flock2, flowMeans, FlowSOM and PhenoGraph. Scatterplots of the software clustering results show how neighbouring clusters from Flock2 and flowMeans were separated with hard straight line boundaries often radiating from a central region (Figure 2B,C and Figure 3B,C), whereas divisions among FlowSOM, PhenoGraph and SPADE3 clusters resembled meandering twisting lines that had echoes of underlying merged sub-clusters (Figure 2D–F and Figure 3D–F). Clusters from SWIFT had softer boundaries, with the fitted Gaussian models visible that slightly overlap each other (Figure 2G and Figure 3G).

### 3.3. Two-Cluster Separation

To assess the performance of software as cell populations come closer together, synthetic two-cluster datasets were generated with multiple replicates at each separation index condition (as described in Section 2.1.1).

**Figure 4:**
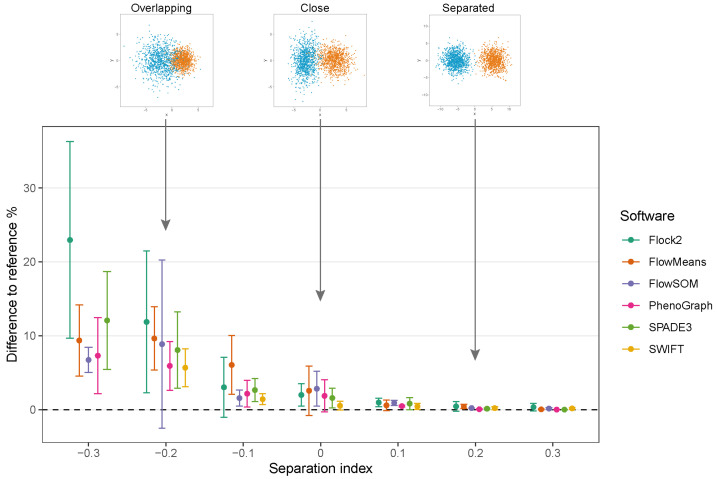
Performance of different software with a two-cluster separation dataset.

While clusters remained separate and distinct with a SI ≥ 0.1, all software outputs were similar to the reference value (differences ranged from 0.01% to 0.97%), and strong repeatability was observed (all standard deviations below 0.8). However, as the two clusters came closer together and the SI approached and fell below 0.0, all six software platforms displayed a decrease in performance; the differences between the software values and the reference value widened, and repeatability deteriorated as demonstrated by the extent of the error bars (Figure 4). The critical SI region appeared to be around −0.1, and any further overlapping of clusters resulted in sharp reductions in software performance and erratic outputs. To place this in the context of real data, the identification of chimeric antigen receptor (CAR)-T cells (e.g., on the basis of the CD19 protein) routinely requires the analysis of less well-separated clusters that fall into this SI region of −0.1 [38]. Overlapping clusters appeared to have the most detrimental effect on Flock2 performance, with differences to the reference value widening from (3.0 ± 4.1)% at SI −0.1 to (11.9 ± 9.6)% at SI −0.2. flowMeans showed similar trends of reduced performance, with difference to reference of (6.1 ± 4.0)% at SI −0.1 and (9.6 ± 4.3)% at SI −0.2 . In contrast, the smaller differences in SWIFT outputs to reference from (1.4 ± 0.73)% to (5.7 ± 2.6)% at SI −0.1 and −0.2, respectively, indicated somewhat better detection of overlapping normally distributed cell populations. However, SWIFT was not able to return two clusters at SI −0.3.

Overall, application of the synthetic two-cluster separation dataset revealed that SWIFT performed better compared to FlowSOM, followed by SPADE3 and PhenoGraph in terms of accuracy and repeatability.

### 3.4. Three-Cluster Separation

Evaluation of the effect of cluster separation on software performance was extended by introducing another cluster to the dataset. The three-cluster dataset added an additional level of complexity as the software now had to make two partitions in the dataset rather than one. Having three clusters also negated issues such as FlowSOM giving a minimum cluster of three for the two-cluster dataset. After causing each software to return three clusters, the number of points per cluster was recorded and the population of cluster 1 was arbitrarily selected to compare against the reference percentage of population value of 50%.

**Figure 5:**
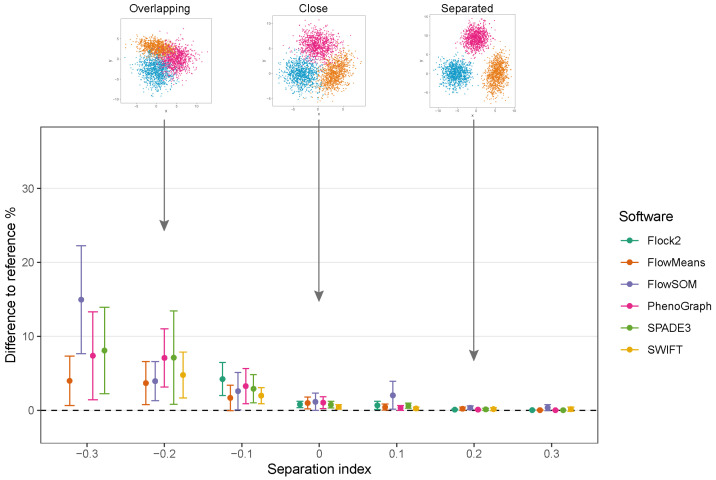
Performance of different software with a three-cluster separation dataset.

The results displayed similar trends in accuracy and precision to the two-cluster dataset (Figure 5). All of the software maintained good accuracy and repeatability at SI ≥ 0, with the exception of FlowSOM at SI 0.1, which displayed lower performance than others. As the SI decreased below 0, software performance again began to deteriorate. The reduction in performance for all software was again particularly noticeable from SI −0.1 to −0.2. Below SI −0.2, the deterioration of performance appeared to plateau for flowMeans, PhenoGraph and SPADE3. Given that it showed consistently smaller differences to the reference value at SI ≥ 0 than other software, flowMeans appeared to be less affected by overlapping clusters; however, whether this was a merit of the software or a consequence of ’random’ equal partitioning of the dataset will require further investigation. Flock2 did not identify three clusters at SI −0.2, and SWIFT at SI −0.3, further highlighting regions of the separation index dataset where clusters became difficult to resolve. Again, it is noted that three-cluster partitioning is prevalent in manual cell analysis.

### 3.5. Skew

To understand whether the behaviours of software were limited to clusters with normal distributions, datasets containing clusters ranging from normal symmetrical to more asymmetrical skewed distributions were generated and processed through the software. Initial runs were performed on skew cluster pairs with a tail-to-tail orientation used here as an exemplar of real flow cytometry data.

**Figure 6:**
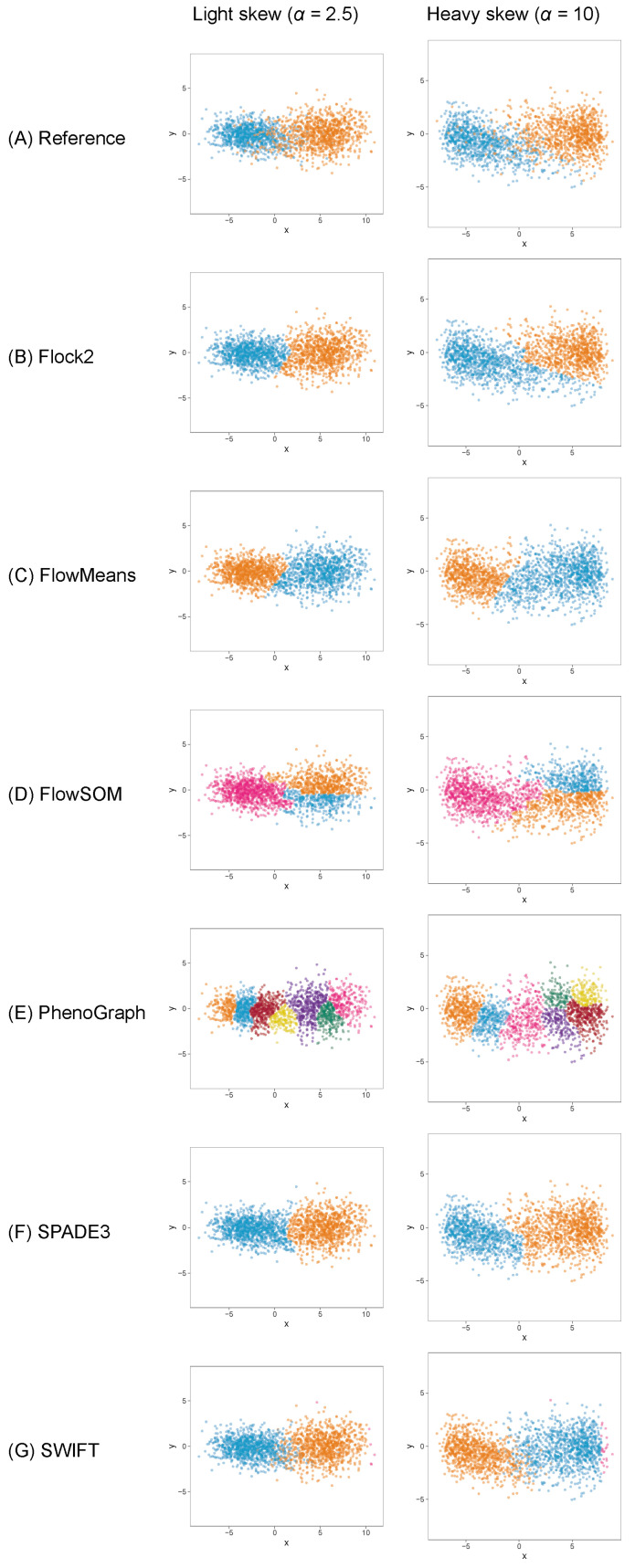
Clustering examples from different software on a two-cluster dataset with skew distributions. Two levels of skew are shown, light skew (α=2.5) and heavy skew (α=10), with cluster orientations all facing tail to tail.

The results showed that, once again, different software returned different clustering outputs and partitioning characteristics from the same dataset (Figure 6). Obtaining the desired output number of clusters, two, was straightforward with Flock2, flowMeans, SPADE3 and SWIFT. FlowSOM gave a minimum output of three clusters, resulting in the appearance of a horizontal bisect of one of the two populations. PhenoGraph outputs partitioned the data into approximately eight clusters as a result of the *k* value that was selected as a compromise between excessive manual intervention and long computational run times (see Supplementary Materials). It is noted that the PhenoGraph algorithm may not be appropriate for analysis of the low-dimensional datasets applied here, however previous work have tested PhenoGraph performance using artificial two-dimensional data [39], and its inclusion in this comparison study here remains useful for users.

**Figure 7:**
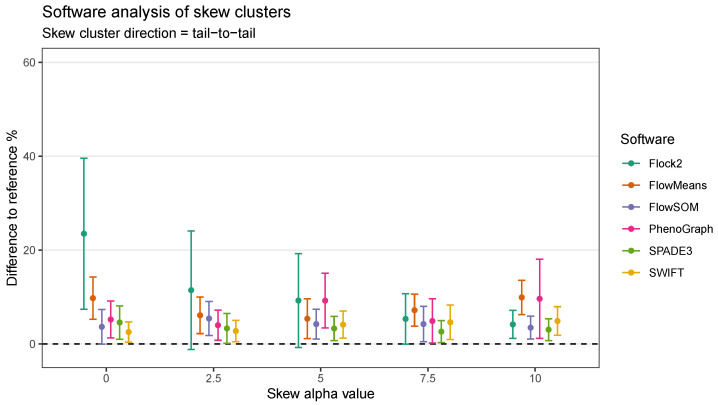
Performance of different software on a dataset with skew cluster orientations facing tail to tail.

Software responded to increasing levels of skew in different ways. In clusters with heavy skew, Flock2, FlowSOM and SPADE3 appeared to partition the data in a more similar manner to the reference dataset compared with flowMeans, PhenoGraph and SWIFT (Figure 6). In this tail-to-tail configuration, Flock2 outputs showed improved accuracy and repeatability as the levels of skew increased, going from a difference to reference of (23.5 ± 16.1)% at no skew (α = 0) to (4.2 ± 3.0)% at heavy skew (α = 10) (Figure 7). The opposite effect was observed for PhenoGraph, with the gap to reference widening from (5.2 ± 3.9)% at skew α= 0 to (9.7 ± 8.4)% at skew α= 10. In comparison, other software outputs showed no significant differences in performance as illustrated in Figure 6. A weak trend was observed for SPADE3 to have better accuracy and repeatability as the level of skew in the datasets increased, and the opposite trend (slight decrease in performance) was observed for SWIFT (Figure 7).

### 3.6. Skew Orientation

**Figure 8:**
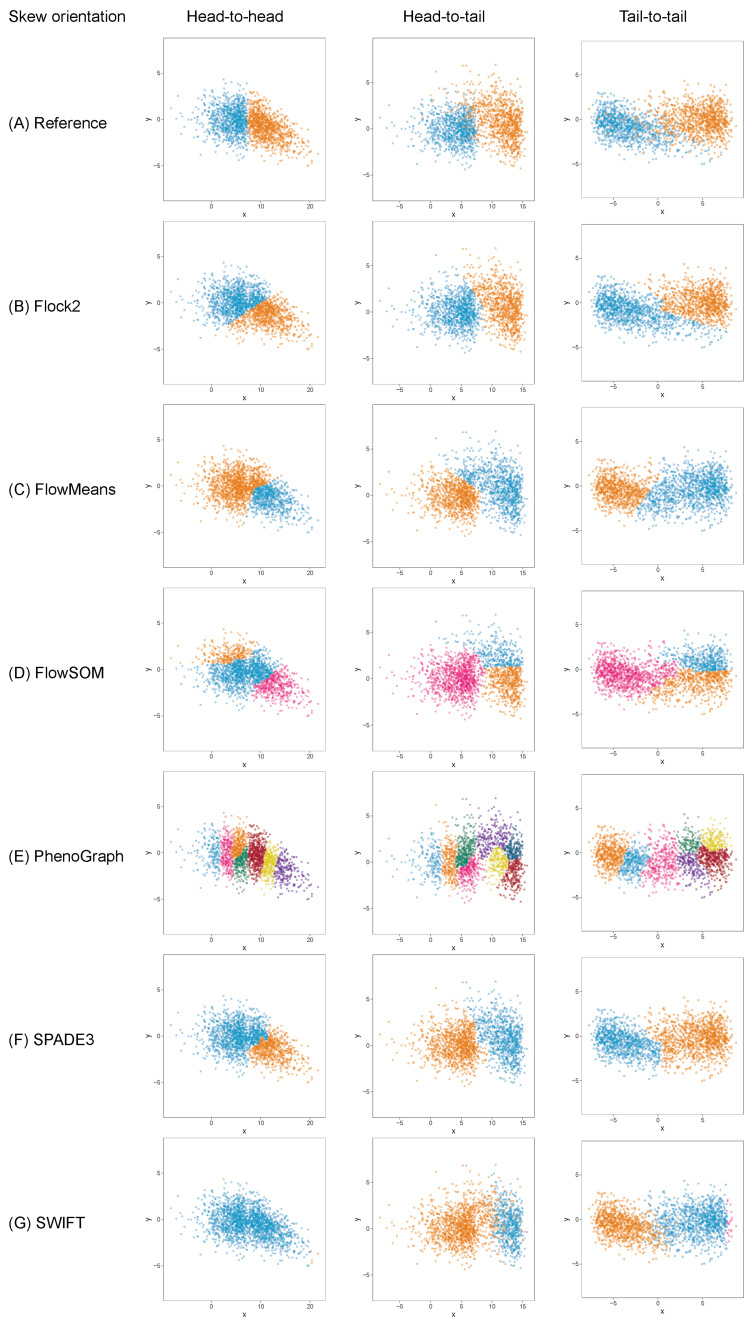
Clustering examples from different software on a two-cluster dataset with skew pairs facing different orientations. All clusters shown with heavy skew (α=10).

It was thought that as well as the level of skewness, the orientation of skew clusters to each other could be a factor affecting a software’s ability to identify cell populations. To investigate this further, the two-cluster skew dataset (initially orientated tail to tail), was extended to include cluster pairs facing both head-to-head and head-to-tail directions (Figure 8). Again, it was seen that whilst most software were able to return two clusters, FlowSOM returned three clusters, and PhenoGraph overclustered the data.

**Figure 9:**
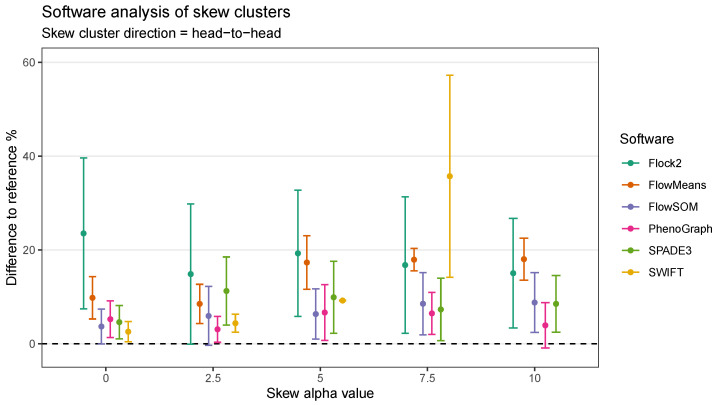
Performance of different software on a dataset with skew cluster orientations facing head to head.

The extension of the skew dataset revealed SWIFT to be the software most affected by skew clusters. In the head-to-head configuration, the gap to reference declined from (2.6 ± 2.2)% at skew α= 0 to (35.7 ± 21.6)% at skew α= 7.5 (Figure 9). Furthermore, SWIFT failed to return any output at skew α=10. The head-to-head pairings also showed flowMeans decreased in performance with increasing skew, with difference to reference going from (9.8 ± 4.5)% at skew α=0 to (18.0 ± 4.5)% at skew α=10.

Comparison across all software suggested that FlowSOM and SPADE3 were least affected by skew distributions, both outperformed Flock2 and flowMeans in terms of accuracy and repeatability.

**Figure 10:**
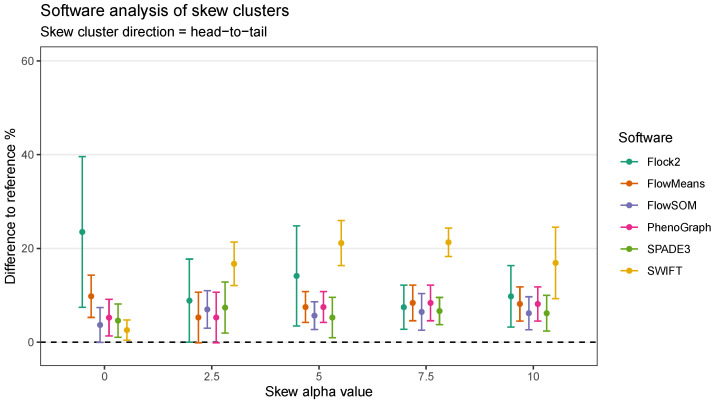
Performance of different software on a dataset with skew cluster orientations facing head to tail.

In the head-to-tail orientation, SWIFT’s performance was noticeably lower than other software at every level of skew above 0 (Figure 10). For instance, the difference to reference of (21.3 ± 3.0)% at skew α=7.5 was worse than the average of all other software (7.5 ± 3.8)%. This suggested that the strategy SWIFT utilises to fit data to Gaussian distributions followed by splitting and merging steps may be challenged by the processing of non-Gaussian distributions.

**Figure 11:**
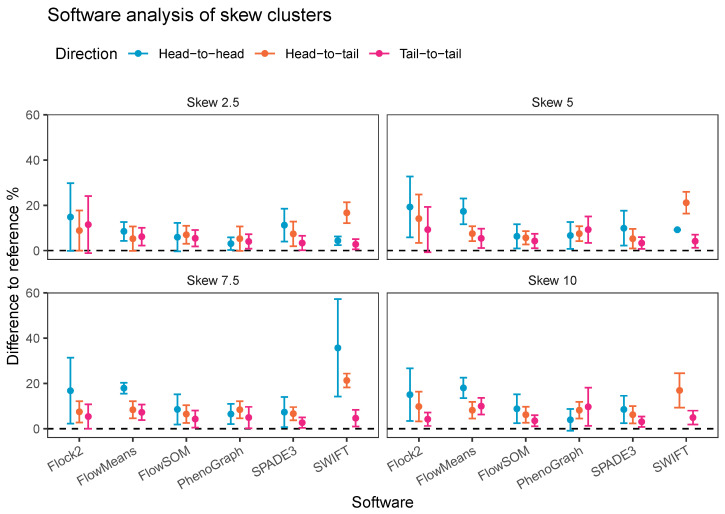
Performance of different software on a dataset with skew cluster orientations facing head to head, head to tail, and tail to tail.

An alternative visualisation of the results from the skew dataset runs suggests that most of the software tested showed a decline in accuracy and repeatability as the orientation shifted from tail to tail, to head to tail and then head to head, respectively (Figure 11). This pattern was generally observed at all levels of skew tested. The changes in performance was likely due to the reduction in the density of events in between the two clusters moving between one orientation to the other, i.e., the higher density of interface events in the head-to-head orientation made data partitioning more difficult. An interesting exception to this pattern was observed with PhenoGraph, where analysis of tail-to-tail skew clusters appeared to slightly reduce in accuracy and repeatability compared with the head-to-head orientated skew clusters. This was possibly because of characteristics of the PhenoGraph algorithm, or more likely that the significant manual intervention required to merge output clusters together to achieve final outcomes artificially improved PhenoGraph results.

Taken together, automated analysis of our synthetic skewed dataset revealed the effects of skew on software performance were largely software dependent, and affected different classes of clustering algorithms in varying ways. Software that model Gaussian distributions onto data were the least well performing (flowMeans and SWIFT). Density-based clustering software appeared to be unaffected by skew characteristics in the data (Flock2). FlowSOM, SPADE3 and PhenoGraph performed well against other software tested here, potentially because they implement overclustering steps that break up the data into smaller populations that each differ in skew properties from the main major population.

## 4. Discussion

Characterisation of ATMPs by automated flow cytometry data analysis software have the potential to improve the quality, repeatability, and robustness of biomanufacturing processes by reducing operator variation as a function of subjective manual gating of clustered data. However, the lack of clarity in how these software derived outputs from inputs, coupled with the absence of toolsets for software validation and standardisation, potentially restricts their implementation by the manufacturing community. In addition, it presents challenges from a clinical and regulatory perspective.

Our previous work on the inter-comparison between synthetic and a real dataset showed clear correlation among cell distribution characteristics examples [21]. Consequently, for this particular cross-platform comparison we are confident that the synthetic data mirrors, to an appropriate level, the key characteristics of low dimensionality cluster data, demonstrates design flexibility and application, and allows for traceable benchmarking (absolute accuracy and repeatability), without the further need to run the platforms through further real data.

In this study, synthetic datasets have been designed and applied to test the performance of six automated flow cytometry cell population identification computational tools. Our use of synthetic datasets with controlled distances between clusters demonstrated similar patterns of behaviours between different software, in which accuracy and repeatability deteriorated as clusters came closer together, particularly below the separation index value of −0.1. These software responses were expected given that overlapping clusters change from multi-modal to unimodal distributions, progressively becoming one large cluster with merged cell populations. The skew datasets implemented here identified considerable variation in outputs between software when processing non-Gaussian distributed clusters, reflecting the different mathematical approaches employed by software to identify cell populations.

Among the six automated tools assessed here, the SWIFT algorithm was found to display better accuracy and repeatability compared to other tools as normally distributed clusters began to overlap and their separation index shifted below 0. However, when assessed further with skewed clusters, SWIFT performance noticeably declined more than others as the skew levels increased. Insights such as these can give operators unfamiliar with computational tools and algorithms a deeper understanding of the potential optimal working ranges of these tools, and the variations in performance that can arise between them depending on the data structures. Furthermore, it could support upstream assay design to ensure data outputs are fit for automated analysis, such as switching to fluorophores leading to more optimised separation, or acquisition settings.

The synthetic dataset approach applied in this study to evaluate automated cell population identification tools extends on, but cannot be directly compared with findings from previous comparison studies, because of the differences in datasets (synthetic and real world) and dataset characteristics used. For example, studies have previously identified FlowSOM as high performing based on high-dimensional datasets [12]; however, in this study, SWIFT outperformed FlowSOM in the low-dimensional, normally distributed dataset, although further testing in the presence of skewed clusters saw SWIFT performance deteriorate.

Compared with previous software comparison studies, the datasets applied here reduce the dependence on narrow cell model examples. Further strengths of this approach include the use of measurable distances between clusters through the separation index, as well as controllable skew parameters, with the benefit of allowing computational tools to be tested one factor at a time, on controlled sets of criteria not feasibly generated from experimental conditions. Of note, the synthetic datasets allow comparisons of software outputs away from subjective manually gated reference values that lack a ‘ground truth’ thus providing explicit statements of accuracy and repeatability.

This study specifically targeted the variation arising from data analysis within the flow cytometry analytical process. Upstream sources of variation from starting materials, sample processing, and instrumentation would require separate comparison studies designed around those factors as variables (e.g., conditions such as lysis, wash, and staining) and with the data analysis software tool kept constant. With regards to the relevance of this data to biological samples analysis, the synthetic datasets here have been designed with essential properties that simulate their equivalent biological counterparts. Therefore, software runs that fail on encountering such data characteristics would directly infer on the (lack of) credibility of results from similar biological samples.

A recognised limitation of this work is that the number of markers simulated is lower than those in real data (usually >3-colour panels) because a priority in this study has been to understand and benchmark how algorithms behave with two or three clusters before introducing further complexities into the datasets. Noting the successful referencing and correlation study we have already completed between synthetic and real data [21], overall, real data have been excluded from this initial research because they are significantly more complex, containing sources of variation from upstream processes and noise components that cannot be controlled to transparently understand the ’black box’ nature of the algorithms investigated. Additionally, it is very difficult to achieve absolute cell counts for real data, so defining measurement accuracy (a critical component of this study) would not be possible. This research here has applied clearly defined synthetic datasets to establish the base functionality of software at lower numbers of parameters before escalating to higher-dimensional datasets (i.e., we cannot run before we can walk). Having achieved this, building more complex datasets is the next key area for further work, and once at that stage, further comparisons between real datasets will illustrate even greater relevance.

The results presented here open up further work to explore more data properties in synthetic dataset design, such as inclusion of more cell populations, higher dimensionality, noise parameters, and in particular, rare cell populations—assessment of which will be a subject for further work within our research. To address the potential for a more heterogeneous cell population mix rather than the homogeneous ones depicted here, further work could model cell subsets within the bulk-component of the skewed population with changing phenotypes (e.g., stem cells undergoing differentiation, T cells in response to cytokine activation), in line with the escalation of various components of complexity within the synthetic dataset design.

Overall, the results of this study suggest that benchmarking of automated flow cytometry software platforms will be possible with a high level of testing integrity using synthetic cluster datasets. The goal of this work was initially to enable biomanufacturers to make better informed decisions about whether or not to implement automated data analysis tools in their workflow instead of/in addition to manual gating methods, based on their own cytometry data—although it is clear that it is also relevant to the clinical community and would potentially impact regulatory science.

Where advanced analysis methods are deemed necessary, the clustering characteristics of different analysis tools illustrated here will facilitate the selection of ones that are fit for purpose. For users, these toolsets can be used to validate and verify installed software and confirm that working ranges match the specifications of their own data. For regulators inspecting process validation documentation, the inclusion of these datasets to provide assurances in automated cell characterisation measurement processes would potentially be desirable. There is potential for the development of synthetic digital reference materials to provide assurances in advanced analytical methods, leading to enhanced measurement confidence in ATMPs.

## Figures and Tables

**Table 1:** Description of computational tools used in this study.

Computational Tool	Description	Reference
Flock2	FLOw Clustering without K; grid-based density clustering algorithm, where the data are divided into hyper-regions, then dense regions are identified, merged and points assigned to their nearest centroids.	[27]
flowMeans	*k*-Means-based clustering that allows multiple clusters to model a single population, with overlapping clusters later being merged.	[29]
FlowSOM	A workflow that reads the data, builds a self-organising map (SOM), builds a minimal spanning tree then computes a meta-clustering output.	[30]
PhenoGraph	Constructs a *k* nearest neighbour graph from high-dimensional data, then uses the Louvain community detection algorithm to partition the graph into sub-populations.	[31]
SPADE3	Spanning-tree progression analysis for density-normalised events; performs deterministic density-dependent downsampling, then *k*-means -based clustering, followed by minimal spanning tree construction. A tree partitioning algorithm aids semiautomated interpretation of data.	[33,34]
SWIFT	Scalable Weighted Iterative Flow-clustering Technique; Gaussian mixture model-based clustering, followed by splitting and merging steps to obtain final clusters that are unimodal but not necessarily Gaussian.	[35,36]

## Data Availability

The data presented in this study are available on request from the corresponding author.

## Supplementary Materials

The following supporting information can be downloaded at: https://www.mdpi.com/article/10.3390/ijms23063224/s1.
