# Supplementary material for: Assessment of Automated Flow Cytometry Data Analysis Tools within Cell and Gene Therapy Manufacturing
Source: Int J Mol Sci. 2022 Mar 17;23(6):3224. doi: 10.3390/ijms23063224 (PMC8955358; doi:10.3390/ijms23063224)
Supplement: Supplementary file 1 [file ijms-23-03224-s001.zip › ijms-1594735-Supplementary Materials.pdf]

Supplementary Materials: Assessment of Automated Flow Cytometry Data Analysis Tools within Cell and Gene Therapy Manufacturing

Melissa Cheung [1,*], Jonathan J. Campbell [2], Robert J. Thomas [1], Julian Braybrook [2] and Jon Petzing [1]

1. Supplementary Methods

1.1. Software runs

The following software were tested: Flock2 [1], flowMeans [2], FlowSOM [3], PhenoGraph [4], SPADE3 [5,6] and SWIFT [7,8].

1.1.1. Flock2

Flock2 analysis was performed on the web-based platform ImmPort Galaxy version 1.2 [9]. FCS files were uploaded to the platform, and converted to a text file using the 'Convert FCS to Text' tool. Flock2 was run using the following user parameters:

- Method: Flock Version 2
- Bins: left blank (auto-select)
- Density: left blank (auto-select)
- Calculate centroids using: Mean Fluorescence Intensity

1.1.2. flowMeans

Initial flowMeans analysis was performed on FlowJo v10 using the R-based plugin included with the installation package. However, later in this research, issues with the plugin began to emerge and after the FlowJo platform failed to deliver results, analysis was switched to the native R platform configuration of flowMeans.

In the FlowJo platform, flowMeans was run using the following user parameters:

- Number of clusters: 3 (values below 3 invalid)

In the R platform, flowMeans version 1.48.0 was run using the following user parameters:

- Number of clusters: 3
- All others: default.

1.1.3. FlowSOM

FlowSOM analysis was performed on the web-based platform ImmPort Galaxy version 1.2 [9]. FlowSOM (Galaxy Version 1.0) was run using the following user parameters:

- Number of expected metaclusters: 3
- Grid size, width: 3
- Grid size, height: 3
- Seed: 42
- Associate each event to: Meta-clusters

The grid size specified the number of clusters, so was changed from the default of 10x10 to 3x3 to force three clusters to be returned. A grid size of 2x2 was found to be an invalid input; two clusters could not be returned.

1.1.4. PhenoGraph

PhenoGraph analysis was performed on the R platform using *Rphenograph* version 0.99.1 [10]. The following parameters were set:

- k, number of nearest neighbours: 150

The output number of clusters could not be specified in PhenoGraph runs. Previous testing on synthetic data with 2,000 points found the default input of $k = 30$ returned outputs of approximately 16 clusters. This output required excessive subjective manual intervention and interpretation to reduce down to two or three clusters, so could not be used. To reduce the output number of clusters, the input k value needed to be increased. However, increasing k until the output reached two or three clusters was not practical because of long run times (>5 hours) required to compute k nearest neighbours of each data point. Subsequent testing showed a starting k value of 150 returned manageable outputs of approximately eight clusters. If the output remained above eight clusters, k was increased by 50 iteratively until the output reached eight clusters or fewer.

It is noted that the PhenoGraph algorithm is intended for clustering of high-dimensional data and not necessarily optimised for analysis of the two-dimensional datasets applied here. From our groups broader collaborations recently and over many years with; big pharma, contract manufacturing organisations, clinical centres, external quality assessment (EQA) centres, international measurement institutes etc, it is very apparent that the availability of (in this instance) flow cytometry data analysis software can often lead to inappropriate application of said software.

This is not unique to biometrology or the biosciences. This behaviour is repeated in other metrology domains and in other industrial sectors. It is a human factors issue and is symptomatic of operators trying to glean further insight from data when not necessarily understanding the boundary conditions and performance criteria of the software tools that are easily and commercially available. It is often the case that available functions within the software solutions can be too comprehensive for tasks at hand. Studies already exist where PhenoGraph performance has been tested using artificial two-dimensional data [11]. Given that PhenoGraph presents an alternative mathematical solution to the other software platforms we have investigated, then it remains useful for users to see the characteristics of its clustering on a basic level compared with other methods, and be better informed about the choice of software solution for their specific data analysis task.

1.1.5. SPADE3

SPADE3 was run within MATLAB R2019a. Selected user input parameters were:

- Ignore compensation
- No transformation
- Local density neighbourhood size: 5
- Local density approximation factor: 1.5
- Maximum allowable cells: 50,000
- Outlier density: 1
- Target density: 20,000 cells
- Algorithm: K-means,
- Number of desired clusters: 100

Each FCS file was run separately with no pooling, noting that all other platforms did not offer pooling. SPADE3 outputs were partitioned into two final populations using the semi-automated partitioning tool, with all suggested partitions being accepted. If three final populations were desired, the larger subpopulation from the first split would be selected for further partitioning.

1.1.6. SWIFT

SWIFT was run within MATLAB R2019a. Selected user input parameters were:

- Input cluster number: 2
- Arcsinh transformation: 0

The SWIFT output number of clusters did not always match the input cluster number, and minimal manual interpretation was sometimes required.

References

1. Qian, Y.; Wei, C.; Eun-Hyung Lee, F.; Campbell, J.; Halliley, J.; Lee, J.A.; Cai, J.; Kong, Y.M.; Sadat, E.; Thomson, E.; et al. Elucidation of seventeen human peripheral blood B-cell subsets and quantification of the tetanus response using a density-based method for the automated identification of cell populations in multidimensional flow cytometry data. *Cytometry Part B: Clinical Cytometry* **2010**, *78B*, S69–S82. doi:10.1002/cyto.b.20554.
2. Aghaeepour, N.; Nikolic, R.; Hoos, H.H.; Brinkman, R.R. Rapid cell population identification in flow cytometry data. *Cytometry Part A* **2011**, *79 A*, 6–13. doi:10.1002/cyto.a.21007.
3. Van Gassen, S.; Callebaut, B.; Van Helden, M.J.; Lambrecht, B.N.; Demeester, P.; Dhaene, T.; Saeys, Y. FlowSOM: Using self-organizing maps for visualization and interpretation of cytometry data. *Cytometry Part A* **2015**, *87*, 636–645. doi:10.1002/cyto.a.22625.
4. Levine, J.H.; Simonds, E.F.; Bendall, S.C.; Davis, K.L.; Amir, E.A.D.; Tadmor, M.D.; Litvin, O.; Fienberg, H.G.; Jager, A.; Zunder, E.R.; et al. Data-driven phenotypic dissection of AML reveals progenitor-like cells that correlate with prognosis. *Cell* **2015**, *162*, 184–197. doi:10.1016/j.cell.2015.05.047.
5. Qiu, P.; Simonds, E.F.; Bendall, S.C.; Gibbs, K.D.; Bruggner, R.V.; Linderman, M.D.; Sachs, K.; Nolan, G.P.; Plevritis, S.K. Extracting a cellular hierarchy from high-dimensional cytometry data with SPADE. *Nature Biotechnology* **2011**, *29*, 886–893. doi:10.1038/nbt.1991.
6. Qiu, P. Toward deterministic and semiautomated SPADE analysis. *Cytometry Part A* **2017**, *91*, 281–289. doi:10.1002/cyto.a.23068.
7. Naim, I.; Datta, S.; Rebhahn, J.; Cavenaugh, J.S.; Mosmann, T.R.; Sharma, G. SWIFT-scalable clustering for automated identification of rare cell populations in large, high-dimensional flow cytometry datasets, Part 1: Algorithm design. *Cytometry Part A* **2014**, *85*, 408–421. doi:10.1002/cyto.a.22446.
8. Mosmann, T.R.; Naim, I.; Rebhahn, J.; Datta, S.; Cavenaugh, J.S.; Weaver, J.M.; Sharma, G. SWIFT-scalable clustering for automated identification of rare cell populations in large, high-dimensional flow cytometry datasets, Part 2: Biological evaluation. *Cytometry Part A* **2014**, *85*, 422–433. doi:10.1002/cyto.a.22445.
9. Bhattacharya, S.; Dunn, P.; Thomas, C.G.; Smith, B.; Schaefer, H.; Chen, J.; Hu, Z.; Zalocusky, K.A.; Shankar, R.D.; Shen-Orr, S.S.; et al. ImmPort, toward repurposing of open access immunological assay data for translational and clinical research. *Scientific Data* **2018**, *5*, 1–9. doi:10.1038/sdata.2018.15.
10. Chen, H. *Rphenograph: R implementation of the phenograph algorithm*, 2015. R package version 0.99.1. Available online: https://github.com/JinmiaoChenLab/Rphenograph (accessed on 2 July 2021).
11. Lorimer, T.; Held, J.; Stoop, R. Clustering: how much bias do we need? *Philosophical Transactions of the Royal Society A: Mathematical, Physical and Engineering Sciences* **2017**, *375*, 20160293. doi:10.1098/rsta.2016.0293.
